# ENdometrial cancer SURvivors’ follow-up carE (ENSURE): Less is more? Evaluating patient satisfaction and cost-effectiveness of a reduced follow-up schedule: study protocol of a randomized controlled trial

**DOI:** 10.1186/s13063-018-2611-x

**Published:** 2018-04-16

**Authors:** Nicole P. M. Ezendam, Belle H. de Rooij, Roy F. P. M. Kruitwagen, Carien L. Creutzberg, Ingrid van Loon, Dorry Boll, M. Caroline Vos, Lonneke V. van de Poll-Franse

**Affiliations:** 1 The Netherlands Comprehensive Cancer Organisation, Godebaldkwartier 419, 3511 DT Utrecht, The Netherlands; 2 CoRPS - Center of Research on Psychology in Somatic diseases, Department of Medical and Clinical Psychology, Tilburg University, Tilburg, The Netherlands; 3 Department of Gynecology and GROW - School for Oncology and Developmental Biology, Maastricht University Medical Center, Maastricht, The Netherlands; 4 Department of Clinical Oncology, Leiden University Medical Centre, Leiden, The Netherlands; 5 Department of Obstetrics and Gynaecology, Amphia hospital, Breda, The Netherlands; 6 Department of Gynecology, Catharina Hospital, Eindhoven, The Netherlands; 7 Department of Obstetrics and Gynaecology, Elisabeth-TweeSteden Hospital, Tilburg and Waalwijk, The Netherlands; 8 Division of Psychosocial Research and Epidemiology, The Netherlands Cancer Institute, Amsterdam, The Netherlands

**Keywords:** Follow-up care, Reduced follow-up, Patient-initiated, Endometrial cancer, Randomized controlled trial, Patient-reported outcomes, Satisfaction with care, Cost-effectiveness

## Abstract

**Background:**

It has often been hypothesized that the frequency of follow-up visits for patients with early-stage endometrial cancer could be decreased. However, studies evaluating effects of a reduced follow-up schedule among this patient group are lacking. The aim of this study is to assess patient satisfaction and cost-effectiveness of a less frequent follow-up schedule compared to the schedule according to the Dutch guideline.

**Methods:**

In this multicenter randomized controlled trial, patients diagnosed in the Netherlands with stage 1A and 1B low-risk endometrial cancer, for whom adjuvant radiotherapy is not indicated (*n* = 282), are randomized. Patients allocated to the intervention group receive four follow-up visits during three years. Patients allocated to the control group receive 10–13 follow-up visits during five years, according to the Dutch guideline. Patients are asked to fill out a questionnaire at baseline and after 6, 12, 36, and 60 months. Primary outcomes include patient satisfaction with follow-up care and cost-effectiveness. Secondary outcomes include healthcare use, adherence to schedule, health-related quality of life, fear of recurrence, anxiety and depression, information provision, recurrence, and survival. Linear regression analyses will be used to assess differences in patient satisfaction with follow-up care between intervention and control group.

**Discussion:**

We anticipate that patients in the intervention arm have a similar satisfaction with follow-up care and overall outcomes, but lower healthcare use and costs than patients in the control arm. No differences are expected in quality-adjusted life-years and satisfaction, but the reduced schedule is expected to be cost-saving when implemented in the Netherlands.

**Trial registration:**

ClinicalTrials.gov, NCT02413606. Registered on 10 April 2015.

**Electronic supplementary material:**

The online version of this article (10.1186/s13063-018-2611-x) contains supplementary material, which is available to authorized users.

## Background

Endometrial cancer is the most common gynecological cancer, with about 1900 newly diagnosed patients per year in the Netherlands. Today, about 20,000 women living in the Netherlands have survived endometrial cancer, of whom almost 8000 were diagnosed in the past five years and are currently receiving follow-up care [1]. Women with early-stage cancer (55%) with favorable features receive surgery only, while those with risk factors or with more advanced stages generally receive adjuvant radiotherapy [2]. Most patients are followed after treatment for five years according to the current guideline, with hospital visits every three or four months in the first two years after treatment, every 4–6 months in the third year, and annually in the fourth and fifth years. Reasons for follow-up include early diagnosis of recurrences—for which curative treatment is available, signaling and treating adverse events of cancer and treatment, psychosocial support, and information provision [3, 4].

The optimal follow-up schedule for patients with endometrial cancer is unknown [4–8]. As a result, guidelines in the Netherlands are consensus-based and do not take risk profile into account. Due to current emphasis on providing high-quality care at lower costs, a critical evaluation of current follow-up practices for cancer patients is needed [9–11]. The transition of follow-up care to the nurse specialist [12, 13] or primary care physician [14, 15] has been presented as a means to increase the cost-effectiveness of follow-up care. However, reduction of the frequency and duration of follow-up visits has not yet been evaluated [4, 10].

Current evidence provides a multitude of reasons that support reduction of standard follow-up visits. First, there is no survival benefit in the detection of asymptomatic recurrences at routine follow-up, compared with symptomatic recurrence or interval detection [4, 5, 8, 16–18], probably because the recurrence rate of early-stage endometrial cancer survivors is low (3%) and because most recurrences (70%) present with symptoms [5]. The majority (70–100%) of the recurrences occur within three years [5]. Second, side-effects from treatments are found in only 6% of the stage 1 patients who received surgery (hysterectomy and salpingo-oophorectomy) alone [2, 19]. Third, follow-up visits evoke distress around the time of the visits [15, 17, 20–22]. Finally, alternative follow-up schedules in other cancer populations do not show decreased patient satisfaction or health-related quality of life (HRQoL) [23, 24]. At the same time, there are reasons to retain some follow-up care, including that follow-up is beneficial for patients for reassurance [15, 21, 25], to provide support for psychosocial, physical, and sexual problems [2, 3, 24], and to provide information [26] and tertiary prevention care [27].

These findings suggest that most early-stage endometrial cancer patients do not need intensive follow-up to detect recurrences, improve survival, or discuss consequences of treatment. However, patients may need *some* follow-up to detect information needs and provide psychosocial counseling. Therefore, a reduced follow-up schedule for low-risk, early-stage endometrial cancer patients should focus on eliminating unnecessary care, which is expected to result in decreased patient worry around visits, prevent wrong patient expectations, and save healthcare costs. At the same time, a minimal number of follow-up visits is needed to support necessary patient counseling and information provision [8–10].

Additionally, it is increasingly recognized that cancer survivors should be provided with information about their disease, treatment, care providers, physical and psychosocial consequences of their cancer and its treatment, care services, and health promotion information [28, 29]. The Institute of Medicine recommends the use of Survivorship Care Plans to provide cancer survivors this information [28]. Although Survivorship Care Plans did not increase satisfaction with information provision and care among gynecological cancer patients [30, 31], they may support symptom monitoring. Adequate monitoring of symptoms by patients would enhance early detection of symptomatic recurrences while limiting the need for follow-up visits.

To obtain evidence on the effects of a reduced follow-up schedule we propose to conduct a nationwide randomized controlled trial (RCT) to study the effects of a reduced follow-up schedule for patients with endometrial cancer. We hypothesized that the patients in the intervention group report at least similar levels of patients’ satisfaction with care at lower costs.

## Methods/Design

### Objectives and hypotheses

The aim of this study is to compare a reduced follow-up schedule of four visits in three years among low-risk, early-stage endometrial cancer survivors, with the schedule according to the current Dutch guideline that includes 10–13 visits in five years. Primary outcomes include patient satisfaction with follow-up care and costs-effectiveness from the healthcare perspective. Secondary outcomes include healthcare use, adherence to the indicated follow-up protocols, reasons for non-adherence, HRQoL, worry including fear of recurrence, anxiety and depression, satisfaction with information provision, healthcare providers’ satisfaction with follow-up schedule, time till recurrence, and survival.

We hypothesize that endometrial cancer patients in the intervention arm of the study are not less satisfied with the follow-up care and do not report worse HRQoL, fear of recurrence, anxiety, depression, and information provision satisfaction. We furthermore hypothesize that healthcare use and associated costs will be lower in the intervention arm, resulting in a cost-effective intervention. More precisely, we expect that by reducing the follow-up schedule from 10–13 to four visits, the costs of these visits will be saved, although some substitution might occur to care by the specialist, specialized nurse, or general practitioner. From a healthcare perspective, we expect this alternative follow-up schedule will save costs.

### Design

A national multicenter (non-inferiority) RCT among 282 endometrial cancer survivors will be conducted. Patients will be randomized 1:1 in the intervention or control group. Since differences in outcomes between groups are expected to be most pronounced within the first three years of follow-up and the largest cost-saving is achieved within three years, we will evaluate this study in a two-step approach, with an evaluation after one, three, and five years. After five years, the follow-up according to the guideline ends. Patients in both arms receive a Survivorship Care Plan, with information on symptoms which would necessitate a consultation.

**Fig. 1:**
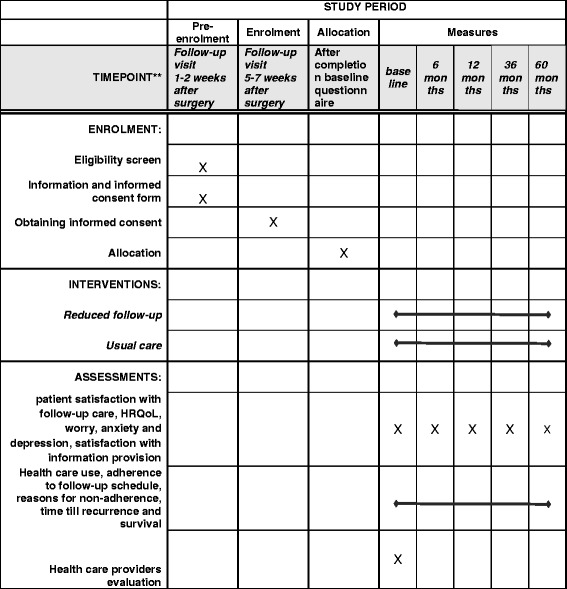
SPIRIT checklist: schedule of enrolment, interventions, and assessments

Doctors and patients cannot be blinded for intervention or control group assignment. A questionnaire will be sent to all patients at baseline (after primary treatment), and 6, 12, 36, and 60 months later. The baseline questionnaire will be assessed before the intervention under study, that is the different follow-up schedules, starts. Healthcare use, recurrences, survival, and costs will be assessed after three and five years. A schedule of the study is presented in Fig. 1. Healthcare professionals will receive a questionnaire at the end of the study. In addition, a non-participation study will be performed registering hospital healthcare use and assessing patient-reported outcomes using a questionnaire. Medical Ethics approval will be obtained before the start of this project.

### Setting

Hospitals throughout the Netherlands can participate. In total, 46 centers (both academic and non-academic centers) will include patients. Participating centers are listed at http://www.ClinicalTrials.gov. Inclusion will take approximately 2.5 years.

### Study population

Low-risk, early-stage endometrial cancer survivors who receive no adjuvant (radio)therapy after initial surgery will be included in the study.

### Inclusion criteria

Patients with endometrioid type endometrial carcinoma with stage 1 (FIGO, 2009) low-risk disease, with the following combination of stage, age, and grade will be eligible: (1) stage 1A, any age, grade 1 or 2; or (2) stage 1B, < 60 years, grade 1 or 2 without lymphovascular space invasion (LVSI). Other inclusion criteria are written informed consent and sufficient oral and written command of the Dutch language. Tumor stage, grade, and type should be histological confirmed by the pathologist before inclusion.

### Exclusion criteria

Exclusion criteria include: (1) any other stage and type of endometrial carcinoma; (2) histological types papillary serous carcinoma or clear cell carcinoma; (3) uterine sarcoma (including carcinosarcoma); (4) receive radiotherapy for current endometrial carcinoma; (5) previous malignancy (except for non-melanomatous skin cancer) < 5 years; (6) having metastases of other tumors; (7) confirmed Lynch syndrome; and (8) previous pelvic radiotherapy**.**

### Recruitment

The national Trial Office of the Netherlands Comprehensive Cancer Organization (IKNL) will organize data collection. Patient-reported outcomes will be obtained by questionnaires [32]. Informed consent will be asked by the treating gynecologist during the postoperative visit. The patient is provided information about the study and an informed consent form. Patients have 2–4 weeks to consider the proposal and can ask questions for instance during an extra visit or a telephone call. In the visit 5–7 weeks after diagnosis, the patient signs the written informed consent and provides address information, in case she is willing to participate. The gynecologist fills out the Randomization Form and sends it to the IKNL Trial Office, who performs the randomization. The patient receives the paper questionnaire and a pre-stamped envelope, to be completed at home. The patients send the completed questionnaire to the IKNL Trial Office. At 6, 12, 36, and 36 months the participant will receive a questionnaire plus pre-stamped envelope at their home address. Non-respondents will be sent a reminder letter and questionnaire within six weeks. Only for the baseline questionnaire will this procedure will be faster to assure a proper baseline measure (reminder through the local principal investigator or research nurse after two weeks). If the patient moves to another hospital, a form will be used to obtain patient data from the new hospital. If the patient does not want to participate in the trial, she is still asked to fill out a single questionnaire and consent to assess healthcare use information, allowing the researchers to compare participants and non-participants. If the patient agrees, the gynecologist completes the registration form for non-participants and sends it to the IKNL Trial Office. The gynecologist provides the patients with a set including a short one-time questionnaire, an informed consent form to assess healthcare use data and a pre-stamped envelope. No other data will be collected for this patient. Questionnaires will be scanned and the database will be checked for a sample of ten patients. On the total database, range and consistency checks will be conducted. Clinical research forms (CRF) will be completed directly in a program.

### Randomization

Patients are randomized via a randomization program, using a computer-generated list of random numbers. Block randomization will be used (no stratification) to assure approximately equal numbers in both groups. Concealment of randomization allocation is guaranteed by the fact that only after written informed consent, the trial manager obtains the randomization allocation from the randomization program and sends it to the gynecologist.

### Intervention versus usual care

#### Usual care

The control group receives follow-up care according to Dutch guideline [2] . This guideline proposes follow-up visits every 3–4 months during the first and second years, every 4–6 months during the third year, and every 12 months during the fourth and fifth years after the end of treatment irrespective of stage and grade [2], resulting in a total of 10–13 visits in five years.

#### Intervention

In the intervention group, the follow-up schedule will be limited to four follow-up visits at 3, 12, 24, and 36 months, under the specific condition that patients have easy and prompt access to care (specialized nurse of gynecologist) if symptoms or questions occur. The content of the follow-up visits will be similar for both groups. In both arms a Survivorship Care Plan, including signs of recurrence, will be provided [33]. This Survivorship Care Plan is personalized based on information that is completed in the randomization form and is emailed as a pdf file to the patient’s caregivers upon randomization.

### Patient and medical outcomes

During the study the following outcomes will be assessed using clinical research forms.

*Patient-related outcomes* include date of birth, other malignancies during the past five years, WHO performance status, weight, height, co-morbidity, menopausal status

*Conduct of preoperative investigations*, including CT or X-ray of the chest, CT or MRI of the abdomen and pelvis, and Ca-125.

*Surgery-related measures* include date of surgery, date of hospital discharge, type of surgery, blood loss, transfusion, complications of surgery, if the patients were in intensive or medium care, if there was a re-intervention for complications

*Pathology investigations* include histology, FIGO stage, FIGO grade, size of the tumor, myometrial invasion, minimal distance between the tumor and the serosa, LVSI.

*Follow-up measures* include date of follow-up visit, being a regular or extra visit, performance status, disease status, healthcare use (gynecologist, oncology nurse), investigations (CT or X-ray of the chest, CT or MRI of the abdomen and pelvis, PET scan, echo, Ca-125)

*Recurrence measures* include symptomatic/asymptomatic recurrence, localization, date, and new treatment.

*End of study measures* include date lost to follow-up, reasons loss to follow-up, and date and cause of death.

*Healthcare use* comprises consults with the specialist, the (specialist) nurse, and the primary care physician, hospital admissions, length of hospital stay, and diagnostics (X-ray, CT, MRI, PET scans, echo, Ca-125).

*Cost-prices* will be obtained from guideline on cost research from the CVZ [34].

### Patient-reported outcomes

We will use existing validated instruments to measure the patient reported outcomes that we hypothesized to be affected by the follow-up visits.

*Patient satisfaction with follow-up care* will be assessed using the Dutch version of the Patient Satisfaction Questionnaire III of which the psychometrics have been assessed in a Dutch oncologic sample [35]. This includes three aspects of healthcare: technical competence (ten items); interpersonal aspects (14 items); and access to care (12 items). The questionnaire can be used as a one-dimensional model, which will be used as the main outcome (PSQ total score).

*Overall quality of life* will be assessed using the EQ-5D [36], a standardized instrument which provides a descriptive profile and a single index value for health status. The measure will be used for the economic evaluation, as it can be used to compute QALYs.

*Cancer-specific HRQoL* will be measured using the EORTC-QLQ-C30 [37]. Much of the content of the questionnaire is appropriate for extended monitoring of health status, including scales assessing physical, role, cognitive and emotional functioning, fatigue and sleep problems, and overall health and QoL.

*Tumour-specific complaints* will be measured using a condition-specific supplement, the EORTC-QLQ-EN24 [25]. The module assesses lymphedema, urological symptoms, gastrointestinal symptoms, body image, sexual/vaginal symptoms, back/pelvic pain, and chemotherapy side effects.

*Worry*, including fear of recurrence, will be assessed using a module from the validated IOCv2 [38] questionnaire. The module consists of six questions including items about worry about the future, worry about health because of the cancer, and worry about a recurrence. This module is a concise measure to assess fear of recurrence and worry.

*Anxiety and depression* will be assessed using the Hospital Anxiety and Depression Scale (HADS), an often used and validated scale in this population [39, 40].

*Satisfaction with information* will be measured using the EORTC-INFO25 module [41]. This questionnaire aims to evaluate the (satisfaction with) information received by cancer patients on different areas of the disease, diagnosis, treatment, and care. The questionnaire contains the following scales: (1) information about the disease; (2) information about medical tests; (3) information about treatment; (4) information about other services—and the following single items: (1) written information; (2) information on CD or tape/video; (3) satisfaction with the amount of information; (4) desire for more information; (5) desire for less information; and (6) helpfulness of information.

*Healthcare use* will also be assessed by asking the frequency of contact with the primary care physician and medical specialist. We will also ask the patient how often these visits were related to cancer. In addition, we will assess how often the patients used additional care services (e.g. psychologist, rehabilitation course, physiologist).

*Co-morbidities at the time of questionnaire completion* will be measured using the Self-administered Comorbidity Questionnaire (SCQ) [42]. Patients will be asked to identify co-morbid conditions developed in the past 12 months. The adapted SCQ lists 14 medical conditions (with the option to list up to three additional conditions).

Additional measures include demographic and socioeconomic variables such as age, education, marital status, and employment status.

### Non-participation evaluation

Patients who do not want to participate in the trial are asked to complete a single questionnaire. Questions include demographics, worry (including fear of recurrence), illness perceptions, and satisfaction with care. For patients who provide informed consent to assess healthcare use, hospital healthcare use (number of visits to the gynecologist and the specialized nurse) will be registered.

### Healthcare provider evaluation

Since the satisfaction of the healthcare provider is also of interest in this project, we will assess the satisfaction of the specialists with the follow-up schemes using a short questionnaire.

### Sample size

The power calculation is performed on the first primary outcome “satisfaction with follow-up care.” The maximal difference between the groups that we find acceptable (non-inferiority margin) is 6 points (< 0.5 SD) on a scale from 0 to 100, with a standard deviation (SD) of 14.3 by [35], based on the rule of thumb as supposed for the EORTC_QLQ-C30 questionnaire [43]. Therefore, with alpha 0.05 and beta 0.80 we need a sample size of 180 (90 per study arm). In this sample size we assume that 30–50 centers will participate and as a consequence we adjusted the number needed in the analysis to account for the clustering of patients within hospitals. With an expected loss to follow-up of 20% and patient who die (16%) over five-year follow-up we need to include 282 patients. Assuming that 60% of the patients will participate, we need 470 eligible patients. In the Netherlands, 450 patients per year meet the inclusion criteria. Since not all hospitals will start inclusion at the same time, we expect an inclusion period of 2.5 years.

### Statistical analysis

Data will be analyzed after one, three, and five years of follow-up. All patients will be included in the analyses and all patients will be analyzed according to the arm to which they were assigned (intention-to-treat). In addition, per protocol analyses will be conducted including patients who received follow-up care as intended in the trial arms. Intention-to-treat and per-protocol analyses are both important in non-inferiority trials. Data will be analyzed using descriptive statistics, linear, and logistic regression analyses. Primary outcome is satisfaction with follow-up care over three-year follow-up. The primary outcome will be analyzed one-sided. Secondary outcomes will also be analyzed as non-inferiority using 0.5 SD as a non-inferiority margin, but will be tested two-sided.

Multilevel multivariate linear and logistic regression analyses will be conducted for continuous and dichotomous outcomes, respectively. Analyses will include relevant pre-defined covariates to improve the power. Differential effects by fear of recurrence will be evaluated by adding the interaction term (group*moderator) to the model. We will carry out repeated measures analyses using multilevel linear mixed models, which accounts for the intra-patient dependency of the repeated measures. Missing outcomes will be assumed missing at random (MAR). An advantage of multilevel linear mixed models is that all patients can be included in the analyses, regardless of whether they have been missing some follow-up measurements. All tests will be considered significant if *p* < 0.05. Clinically meaningful differences will be determined with Norman’s “rule of thumb,” whereby a difference of > 0.5 SD indicates a threshold of discriminant change in health status scores of a chronic illness [43]. If we have the baseline data of a patient, we will include this patient in the analysis.

The cost-effectiveness analysis will be performed from a healthcare perspective with a time horizon of 36 months and will be expressed as the incremental cost per QALYs (based on the EQ-5D) and the incremental costs per satisfied patient. Based on results up to 36 months, five-year cost-effectiveness results will be estimated. Patient adherence to the reduced follow-up schedule will explicitly be addressed in the cost-effectiveness analysis. A budget impact analysis will be performed according to the ISPOR guidelines [44]. The budget impact analysis addresses the financial stream of consequences related to the implementation of and compliance with the reduced follow-up scheme to assess affordability. The budget impact will depend on both the cost-savings due to reduced follow-up visits, the uptake by specialists, adherence of patients as well as potential cost increases, e.g. at the level of the primary care physician.

Results of the trial will be published in scientific journals and communicated to the patients via patient organizations.

## Discussion

Although the current guidelines in the Netherlands describe five years of hospital-based follow-up for women after their treatment for endometrial cancer, hardly any scientific evidence exists to support this timely and expensive practice which involves increasing resources due to the growing number of cancer survivors. Therefore, a critical evaluation of follow-up care for endometrial cancer survivors is needed. The current landscape of evidence predominantly focuses on transitioning follow-up care to nurses or primary care physicians [45]. However, we hypothesize that reducing the frequency of follow-up care would result in a higher cost-effectiveness through retaining levels of satisfaction and outcomes, while decreasing the costs of follow-up care. Outcomes of our trial may therefore evoke a new framing of the issue of follow-up care, by shifting the focus from transitioning care to eliminating unnecessary care, without disregarding a minimal level of follow-up care that is needed to provide adequate information, psychosocial support, and tertiary prevention [15, 21, 25]. Although Survivorship Care Plans did not increase satisfaction with information and care in a previous trial [30, 31], we expect that Survivorship Care Plans support symptom monitoring, thereby limiting the need for additional follow-up visits to detect symptomatic recurrences.

Patients who do not agree to participate in the trial may have higher needs regarding follow-up care and, for instance, higher levels of psychological distress. If our non-participation study shows differences between participants and non-participants, the results of the study may not be generalizable to the full population of interest. In light of this potential result, individualized follow-up care may be a viable solution, by tailoring the number of follow-up visits based on patient preferences and (clinical) characteristics. On the other hand, if participants of the trial seem representative of the target population and a reduced follow-up schedule results in a similar patient satisfaction at lower costs, the current guideline will be adapted and the reduced schedule will be implemented throughout the Netherlands. Furthermore, a reduced follow-up schedule may be applicable to other cancer types and practices outside of the Netherlands.

Other trials in this field that have been published, including the ENDCAT trial, focus on transitions to (nurse-led) telephone follow-up [12, 13]. Ongoing trials include OPAL, TOPCAT-G, and TOTEM [4]. Similar to ENSURE, OPAL (trial number NCT01853865) is comparing a reduced patient-initiated follow-up compared to standard care and restricted recruitment to low- and intermediate-risk endometrial cancer. TOPCAT-G (trial number ISRCTN45565436) assesses a reduced regime of nurse-led telephone follow-up and includes non-endometrial gynecological cancers but excludes sarcomas and trophoblastic tumors. Unlike others, TOTEM (trial number NCT00916708) has an active arm for more intense follow-up and is uniquely powered to compare survival among endometrial cancer patients.

In conclusion, the ENSURE trial will provide new insights into the (cost-)effectiveness of a reduced follow-up schedule in low-risk endometrial cancer patients and will guide future recommendations for evidence-based follow-up. Results will show whether patients are willing to participate in the trial and thus agree on reduced follow-up. In addition, the non-participation study will provide insight why some patients do not agree to be randomized in one of both study arms. If reduced follow-up results in similar levels of satisfaction and outcomes at lower costs, a reduced schedule can become part of regular follow-up care for early-stage endometrial cancer patients.

## Trial status

At the time of manuscript submission, the trial was open for patient inclusion. Additional information on the trial can be found in Additional files 1 and 2.

## Additional file

Additional file 1: Table WHO Trial Registration Data Set (TRDS) and additional protocol information of the ENSURE trial. (DOCX 50 kb)

Additional file 2: SPIRIT 2013 Checklist: Recommended items to address in a clinical trial protocol and related documents*. (DOC 121 kb)

## Abbreviations

CRF: Clinical research forms
ENSURE: ENdometrial cancer SURvivors’ follow-up carE
HADS: Hospital Anxiety and Depression Scale
HRQoL: Health-related quality of life
IKNL: Netherlands Comprehensive Cancer Organization
MAR: Missing at random
PSQ: Patient Satisfaction Questionnaire
QALY: Quality-adjusted life-years
RCT: Randomized controlled trial
SCQ: Self-administered Comorbidity Questionnaire
SD: Standard deviation
