# Supplementary material for: ENdometrial cancer SURvivors’ follow-up carE (ENSURE): Less is more? Evaluating patient satisfaction and cost-effectiveness of a reduced follow-up schedule: study protocol of a randomized controlled trial
Source: Trials. 2018 Apr 16;19:227. doi: 10.1186/s13063-018-2611-x (PMC5902894; doi:10.1186/s13063-018-2611-x)
Supplement: Supplementary file 1 — Table WHO Trial Registration Data Set (TRDS) and additional protocol information of the ENSURE trial. (DOCX 50 kb) [file 13063_2018_2611_MOESM1_ESM.docx]

**Table WHO Trial Registration Data Set (TRDS) and additional protocol information of the ENSURE trial**

| Primary Registry and Trial Identifying Number | Trial Registration: http://www.ClinicalTrials.gov. Identifier: NCT02413606 |
| --- | --- |
| Date of Registration in Primary Registry | registered April 10, 2015 |
| Secondary Identifying Numbers | Protocol version: 1.5  Medical Research Ethics Committee Reference Number: METC Brabant 2015.011.  Grant Reference Number Dutch Cancer Society: IKZ 2014-6677 |
| Source(s) of Monetary or Material Support | Dutch Cancer Society |
| Primary Sponsor | Netherlands comprehensive cancer organisation |
| Secondary Sponsor(s) | - |
| Contact for Public Queries | Nicole P.M. Ezendam, PhD The Netherlands Comprehensive Cancer Organisation (IKNL) Zernikestraat 29, 5612 HZ, Eindhoven, the Netherlands Email: N.Ezendam@iknl.nl; Telephone: +312346096 |
| Contact for Scientific Queries | Nicole P.M. Ezendam, PhD The Netherlands Comprehensive Cancer Organisation (IKNL) Zernikestraat 29, 5612 HZ, Eindhoven, the Netherlands Email: N.Ezendam@iknl.nl; Telephone: +312346096 |
| Public Title | Nazorg bij patiënten met baarmoeder kanker |
| Scientific Title | ENdometrial cancer SURvivors’ follow-up carE (ENSURE): Less is more? Randomized controlled trial to evaluate patient satisfaction and cost-effectiveness of a reduced follow-up schedule |
| Countries of Recruitment | Netherlands |
| Health Condition(s) or Problem(s) Studied | Endometrial cancer |
| Intervention(s) | Patients allocated to the intervention group receive 4 follow-up visits during 3 years. Patients allocated to the control group receive 10-13 follow-up visits during 5 years, according to the Dutch guideline. |
| Key Inclusion and Exclusion Criteria | Inclusion criteria:  Endometrioid type endometrial carcinoma with stage 1 (FIGO, 2009) With the following combination of stage, age and grade will be eligible: 1) stage 1A, any age, grade 1 or 2, or 2) stage 1B, < 60 years, grade 1 or 2 without LVSI.  Written informed consent  Sufficient oral and written command of the Dutch language.  Exclusion criteria  1) any other stage and type of endometrial carcinoma, 2) histological types papillary serous carcinoma or clear cell carcinoma, 3) uterine sarcoma (including carcinosarcoma), 4) receive radiotherapy for current endometrial carcinoma, 5) previous malignancy (except for non-melanomatous skin cancer) < 5 years, 6) having metastases of other tumors, 7) confirmed Lynch syndrome, and 8) previous pelvic radiotherapy. |
| Study Type | Randomized controlled trial, with two arms  Doctors and patients cannot be blinded for intervention or control group assignment.  Concealment of randomization allocation is guaranteed by the fact that only after written informed consent, the trial manager obtains the randomization allocation from the randomization program and sends it to the gynecologist. |
| Date of First Enrollment | 30 September, 2015 |
| Sample Size | 282 patients needs to be included and questionnaire data needs to be obtained. |
| Recruitment Status | Recruitment completed March 5th 2018 |
| Primary Outcome(s) | Primary outcome is satisfaction with follow-up care over three year follow-up and cost-effectiveness from the health care perspective.  *Patient satisfaction* *with follow-up care* will be assessed using the Dutch version of the Patient Satisfaction Questionnaire III. This includes three aspects of health care: technical competence (10 items), interpersonal aspects (14 items), and access to care (12 items). The questionnaire can be used as a one-dimensional model, which will be used as the main outcome (PSQ total score). |
| Key Secondary Outcomes | Secondary outcomes include health care use (number of visits and contact with the hospital), adherence to schedule, health-related quality of life (EORTC QLQC30), fear of recurrence (worry scale from the impact of cancer questionnaire), anxiety and depression (HADS), information provision (INFO25), recurrence, survival. |
| Ethics Review | Approved, 15 January 2015  Medical Research Ethics Committee Reference Number: METC Brabant 2015.011.  Hilvarenbeekseweg 60, 5022GC Tilburg, info@metcbrabant.nl |
| Completion date | Expected in March 2023 |
| Summary Results | No results available currently |
| IPD sharing statement | The data that support the findings of this study are available from PROFILES but restrictions apply to the availability of these data, which were used under license for the current study, and so are not publicly available. Data are however available from the authors upon reasonable request and with permission of PROFILES. |
| Data collection and procedures | Data collection will be done by the Trial Buearu of the Netherlands Comprehensive Cancer Organisation. No additional auditing of the trial conduct will be conducted. Personal information of participants remain in the hospital. Investigators have access to quasi-anonymized data. |
| Monitoring | Independent local data managers will check written informed consent and eligibility, before obtaining additional information from the patients’ medical files, being a first quality check on the data. In addition, monitoring of this low-risk trial will be done by qualified monitors according to the NFU guidelines [NFU Kwaliteitsborging mensgebonden onderzoek 2.0, 2012]. This monitoring will be performed by independent monitors of the trial bureau, monitoring written informed consent, eligibility, CRFs, procedures, inclusion and loss-to-follow up. |
| Communication of protocol amendment | Protocol amendments will be communicated to the Medical Ethics Committee and all local investigators and if relevant to the participants. |
